# Strategies of cell and cell-free therapies for periodontal regeneration: the state of the art

**DOI:** 10.1186/s13287-022-03225-z

**Published:** 2022-12-27

**Authors:** Xiuting Wang, Jinlong Chen, Weidong Tian

**Affiliations:** 1 State Key Laboratory of Oral Diseases & National Clinical Research Center for Oral Diseases, West China School of Stomatology, Sichuan University, Chengdu, People’s Republic of China; 2 National Engineering Laboratory for Oral Regenerative Medicine, West China School of Stomatology, Sichuan University, Chengdu, People’s Republic of China; 3 Department of Oral and Maxillofacial Surgery, West China Hospital of Stomatology, Sichuan University, Chengdu, 610041 People’s Republic of China

**Keywords:** Periodontal regeneration, Tissue engineering, Endogenous regeneration, Biomimetic design, Extracellular matrix

## Abstract

**Background:**

Periodontitis often causes irrevocable destruction of tooth-supporting tissues and eventually leads to tooth loss. Currently, stem cell-based tissue engineering has achieved a favorable result in regenerating periodontal tissues. Moreover, cell-free therapies that aim to facilitate the recruitment of resident repair cell populations to injured sites by promoting cell mobilization and homing have become alternative options to cell therapy.

**Main text:**

Cell aggregates (e.g., cell sheets) retain a large amount of extracellular matrix which can improve cell viability and survival rates after implantation in vivo. Electrostatic spinning and 3D bioprinting through fabricating specific alignments and interactions scaffold structures have made promising outcomes in the construction of a microenvironment conducive to periodontal regeneration. Cell-free therapies with adding biological agents (growth factors, exosomes and conditioned media) to promote endogenous regeneration have somewhat addressed the limitations of cell therapy.

**Conclusion:**

Hence, this article reviews the progress of stem cell-based tissue engineering and advanced strategies for endogenous regeneration based on stem cell derivatives in periodontal regeneration.

## Introduction

The periodontium is an intercalated structure of hard and soft tissues surrounding and supporting the teeth and is capable of resisting and dispersing masticatory forces in a highly hierarchical manner. The periodontium consists of four different tissues: the gingiva, cementum, periodontal ligament (PDL), and alveolar bone [1, 2]. Periodontitis is the most common reason for the destruction of periodontal tissue, leading to the loss of teeth in serious situations, and it significantly decreases mastication function and quality of life [2, 3]. Conventional treatments include supragingival cleaning, subgingival scaling, and root planning to remove pathogenic factors [4], which only halt the further development of inflammation and do not work to restore damaged periodontium. In recent decades, bone grafting and guided tissue/bone regeneration techniques have been widely used in the clinic and can achieve some periodontal regeneration; however, the results are unpredictable and limited.

Tissue engineering plays a significant role in regenerative medicine and consists of three basic elements: cells, scaffolds, and growth factors. Of these, stem cells are crucial given their ability to self-renew and differentiate into a variety of cell types. Many animal models and clinical studies [5, 6] have confirmed that stem cell-based tissue engineering has achieved a favorable result in regenerating periodontal tissues. Nevertheless, its clinical application is hampered by various issues, such as stem cell acquisition, in vitro expansion, immunogenicity, and ethics. The phenomenon of the native wound healing cascade hints that it is possible to harness the intrinsic regenerative potential of endogenous tissues for tissue repair and regeneration without the aid of exogenous stem cells [7]. However, naturally endogenous regenerative processes are generally limited and unable to successfully regenerate many tissues. Therefore, current strategies for endogenous regenerative medicine aim to facilitate the recruitment of resident repair cell populations to injured sites by promoting cell mobilization and homing and have become alternative options to cell therapy.

**Fig. 1:**
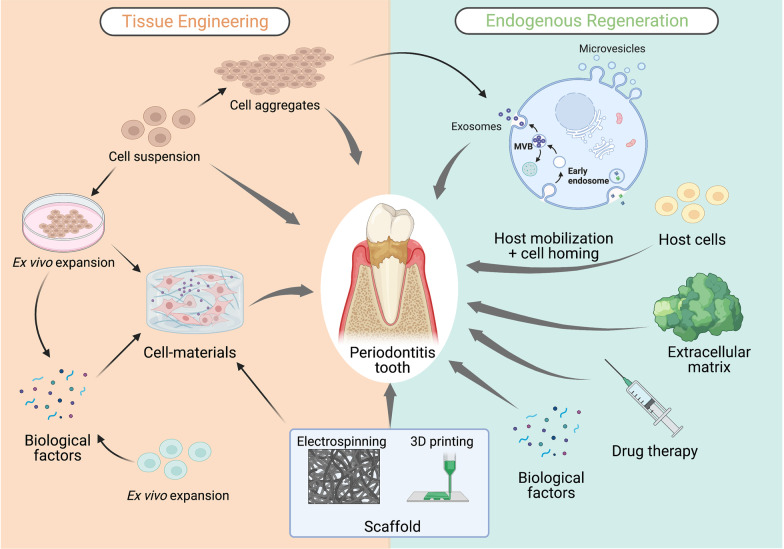
Schematic of periodontal regeneration. Cell-based tissue engineering and cell-free endogenous regeneration in periodontal regeneration. Created with BioRender.com

Hence, this paper will review the progress of cell-based tissue engineering and cell-free endogenous regeneration applied to periodontal regeneration (a schematic of periodontal regeneration is shown in Fig. 1) and discuss the most likely means of achieving functional periodontal tissue regeneration.

### Tissue engineering and periodontal regeneration

Mesenchymal stem cells (MSCs) originate from the mesoderm and possess immunomodulatory and anti-inflammatory functions as well as the ability to regulate intercellular communication and function via paracrine secretion [8], which makes them the most commonly used stem cells in current research for the study and treatment of human refractory diseases [9]. Greater than 1300 clinical trials have utilized MSCs, and these trials are currently listed at www.clinicaltrials.gov.

Advances in MSCs-based tissue engineering have given rise to novel therapeutic strategies for tooth and periodontal repair and regeneration. Stem cells applied to periodontal regeneration can be classified as dental stem cells and nondental stem cells. Dental stem cells include dental pulp stem cells (DPSCs), PDL stem cells (PDLSCs), dental follicle cells, stem cells from human exfoliated deciduous teeth (SHED), stem cells from the apical papilla, and gingival MSCs (GMSCs) [10, 11]. Among these, PDLSCs [12–14] are the most commonly used in periodontal tissue regeneration given their multilineage differentiation potency, anti-inflammation properties, and immunomodulation properties [15]. Nondental stem cells include embryonic stem cells, induced pluripotent stem cells, bone marrow MSCs (BMMSCs), and adipose-derived stem cells. BMMSCs [16–18] have been reported to differentiate into PDL, have a strong osteogenic differentiation ability and are readily available, allowing bone marrow to become one of the main stem cell sources for regenerating the periodontium.

#### Cell aggregates and periodontal regeneration

Transplanting an adequate number of seed cells with optimal viability into the defects and guaranteeing that they remain functional is the first step toward achieving clinical efficacy for cell therapies. Therefore, it is essential to choose an appropriate method of cell transplantation. When administered as a suspension into the area of periodontal defects, there is little time for the cells to adhere, which results in low cell survival and carries the risk of cell migration to other parts of the body [19].These deficiencies are ameliorated by cell aggregates [20, 21], which are a cluster of cells bound to each other through cell proliferation and/or cell aggregation and are valuable for studying tissue development [22]. Various studies have shown enhanced cellular function in cell aggregates compared to that in single-cell suspensions, such as immunomodulatory potency, multilineage differentiation of MSCs aggregates, and improved cell viability and survival rates after implantation in vivo [23]. Cell aggregates can be produced by a variety of methods; among them, the cell sheet technique is the most frequently used method in periodontal regeneration. Cell sheets [24] retain a large amount of extracellular matrix (ECM), which provides a microenvironment for cell survival, assists in maintaining cell stemness, enhances cell viability, promotes cell adhesion to damaged areas, and stimulates host cell homing. The most popular approach to harvesting cell sheets is via temperature-responsive intelligent polymer cell culture dishes. After lowering the temperature below 32 °C, the polymer dish surfaces become hydrophilic; as a result, an intact cell sheet can be separated from the dish surface without enzymatic treatment [25].

PDLSCs sheets could form in these temperature-responsive culture dishes and achieve ectopic regeneration of cementum-like and PDL-like tissues after transplantation [26]. However, only a thin layer of cell sheet was insufficient for the regeneration of large periodontal defects. One study added 50 ug/mL ascorbic acid to the cell culture medium, prolonged the culture of cells for 14 days, and reported an increase in secreted ECM proteins, such as type I collagen, laminin and fibronectin [27]. Raju et al. [28] fabricated a three-dimensional (3D) composite cell sheets containing 10 layers of cells. Only the complex cell sheet, combined with the functional linkage of PDL-like fibers to the tooth root, was shown to physically renew the bone-ligament structure in ectopic and orthotopic transplantation. Each cell type derived from a different tissue has a characteristic ability to differentiate, so the combination of stem cells from distinct tissue sources could produce enhanced periodontal regeneration outcomes. Zhang et al. [29] combined PDLSCs with BMMSCs, significantly enhancing the expression of bone- and ECM-related genes and proteins. To address problems, such as necrosis and inflammation of the transplanted tissue, a previous study mixed PDLSCs and human umbilical vein endothelial cells (HUVECs) for transplantation, which led to the formation of PDL-like tissues in vivo and higher numbers of blood vessel lumens [30]. In addition, numerous studies have further improved regeneration by adding bioactive molecules or bone graft materials to enhance the properties and mechanical strength of the cell sheets.

#### Scaffold materials

For sophisticated and variable periodontal regeneration microenvironments and large-scale periodontal defects, in addition to the use of cell sheets, researchers are turning their attention to well-designed scaffold materials that mimic periodontal structures to promote 3D periodontal regeneration. There are different types of scaffold materials (Summarized in Table 1) [31]. Natural biomaterials, such as collagen and chitosan, possess good biocompatibility and low immunogenicity, and chitosan [32] also has favorable osteoinductive, antibacterial, and anti-inflammatory properties. Bioceramic materials [33], such as hydroxyapatite (HA), β-tricalcium phosphate (β-TCP), and bioactive glass, have been extensively used to bolster the healing of alveolar bone in the periodontium. Bioceramic scaffolds typically provide high mechanical stability with outstanding osteoconductive and osteoinductive properties and are suitable for alveolar bone regeneration. Synthetic polyester-based polymers [31], such as polylactic acid, polyglycolic acid, poly(lactic-co-glycolic acid) (PLGA), and polycaprolactone (PCL), have many unique advantages, including highly tunable physicochemical properties, controlled biodegradation rates, and a simple and straightforward manufacturing process that allows for mass production. They are therefore commonly used as periodontal scaffold materials. It should be noted that the degradation byproducts of polyesters can be toxic but are considered safe for this application due to the minimal number of residual particles being released at a very slow rate. A scaffold, as a delivery vehicle for cells and bioactive molecules and as a means to supply space for tissue growth, can be fabricated into diverse structural forms by different manufacturing techniques. Hydrogels, microspheres, micropatterns, and monolayer or multilayer scaffolds are each manufactured differently and have their own unique merits. Generally, previous studies focused on promoting tissue generation by combining different scaffolding materials or by adding active factors. However, currently, the fabrication strategy of scaffolds has moved from [34] to multiphasic constructs [35–39] with biomimetic periodontal tissue to address the highly hierarchical structure of the native periodontium, in which electrospinning and 3D bioprinting technologies show tremendous potential.

**Table 1:** A summary of the diverse types of scaffold materials

Types of materials	Including examples	Advantages	Disadvantages	References
Natural biomaterials	Collagen and chitosan	Good biocompatibility and low immunogenicity	Easily degraded and poor mechanical properties	[31, 32]
Bioceramic materials	HA, β-TCP and bioactive glass	High mechanical stability with outstanding osteoconductive and osteoinductive properties	High fragility, slow degradation rate and inferior workability	[33]
Synthetic polyester-based polymers	Polylactic acid, polyglycolic acid, PLGA, and PCL	Highly tunable physicochemical properties, controlled biodegradation rates, and a simple and straightforward manufacturing process	Hyproducts of polyesters can be toxic but are considered safe for this application	[31]

#### Electrospinning technology and periodontal regeneration

Electrospinning technology [40, 41] increases the protein content through controlled fiber diameter, porosity, and ideal morphology. Better surface properties may activate some related acellular signaling and specific gene expression, stimulate cellular responses and are appropriate for the architecture of orderly aligned periodontal fibers and random mineralized matrix accumulation to form alveolar bone. With rat PDLSCs, Shang et al. [41] created electrospun scaffolds with two distinct orientations as an example of in vitro electrospun contact guiding. Contrary to randomly arranged fibers and a porosity control, where the cells took on polygonal or random geometries, the parallel and cross-aligned scaffolds showed elongated morphologies along the direction of fiber alignment. By inserting highly aligned biodegradable poly(-caprolactone)-poly(ethylene glycol) (PCE) copolymer electrospun nanofibrous mats into porous chitosan, Jiang et al. created a 3D multilayered scaffold. The aligned nanofiber-embedded scaffold guided arrangement and enhanced cell behavior in vitro. In vivo, it showed a more orderly arrangement of regenerated PDL, a more extensive formation of mature collagen fibers, and an increase in periostin expression compared to those of the other two groups.

To increase osteoconductivity, improve cellular and tissue interactions, and improve vascular growth, a biphasic scaffold was built by attaching a fused deposition modeled bone compartment coated with a calcium phosphate (CaP) layer to a melt electrospun periodontal compartment comprising larger pores. In vivo, increased bone formation, more pronounced vascularization, and greater periodontal attachment on the dentin surface were observed [43]. Furthermore, some bioactive components, such as chitosan [32], bioceramics [44] and functional polymers [45], can be easily blended into the electrospun nanofiber matrix to modulate its physical, chemical, and biological properties and its regenerative capacity. Although the histologic sections provided in Jiang et al.’s study showed good attachment and orientation of the newly formed PDL, the presence of slowly degrading PCE electrospinning material was not evident [42]. This finding may indicate that the scaffold material was moved during implantation or was only partially located at the periphery of the defect and was not necessarily placed exactly in its central portion. Limitations of electrospinning include that it is slow, costly, difficult to control, and does not yield a wide range of scalable pattern geometries. In contrast, 3D printing, with rapid production and low material wastage, has been demonstrated to be an effective approach to reconstructing periodontal tissue.

#### 3D printing and periodontal regeneration

3D printing [46] is a method of additive manufacturing where a 3D object can be created by adding material layer by layer. It has been applied to reconstruct and regenerate organs and tissues. For irregular periodontal defects caused by periodontitis, 3D bioprinting has the benefit of being able to realize structures with specific alignments and interactions [47, 48]. Utilizing dental stem cells, Lee et al. [36] developed a region-specific scaffold with three stages of microstructures that was preoptimized for the regeneration of cementum, PDL, and alveolar bone. In 2015, Rasperini et al. [49] evaluated the first clinical case of applying a 3D-printed scaffold to a patient with a periodontal defect. Specifically, 3D-printed PCL scaffolds containing recombinant human platelet-derived growth factor-BB (rhPDGF-BB) were transplanted in single-walled bone defects in patients. The transplanted scaffolds were then exposed intraorally at 13 months. A 3-mm increase in adhesion and 75.9% stent retention were noted at 14 months with predominantly connective tissue healing and minimal evidence of bone repair. Although clinical outcomes need further improvement, the use of 3D-printed scaffolds with different spaces for PDL and alveolar bone is a worthwhile endeavor. To boost the degradation of PCL, Peng et al. [50] mixed PCL with PLGA in different ratios and found that the increase in the PLGA ratio significantly accelerated the degradation of the hybrid scaffold, smoothed the surface, and increased wettability. Furthermore, human PDLSCs (hPDLSCs) showed significantly increased adhesion, proliferation, and osteogenic capacity at 0.5 PCL/0.5 PLGA.

Advances in material technology have made 3D bioprinting possible, allowing cells to be blended into bioink and positioned in certain locations in the printed tissue, increasing the accuracy of the structural details of the inner and outer layers [51–53]. Several studies have been conducted to explore various bioprinting materials and optimize printing conditions using hPDLSCs [51, 52]. These studies revealed that adjusting the composition and concentration of the materials helped to improve cell viability and diffusion and to increase cell-material interactions, which promoted tissue regeneration [51, 52]. Tian et al. [51] printed sodium alginate (SA)/gelatin (Gel)/nano-HA (na-HA) composite bioscaffolds by extrusion 3D bioprinting technology and subsequently used hPDLSCs and composite hydrogels as bioinks to print SA/Gel/na-HA/hPDLSC cellular bioscaffolds. The results showed that the SA/Gel/na-HA composite hydrogels exhibited good rheological properties and a high swelling rate suitable for the printed scaffolds, which indicated that they had sufficient porosity. The compression properties of the composite bioscaffolds were also improved by the addition of na-HA. In another study, Li et al. [54] mixed hPDLSCs into bioink (1 × 10^7^ cells/mL) and printed PDL layers on the surface of 3D-printed titanium scaffolds. Scanning electron microscopy revealed that the hPDLSCs were neatly arranged and aligned in the cell printing group compared to the cell seeding group. On Day 7 of culture, Cementum protein (CEMP1) expression in the cell printing groups was significantly higher than that in the cell seeding groups. This finding indicates that the cell printing method is more reliable than cell seeding. HE staining at 6 weeks showed new bone formation in the porous scaffold of the cell seeding group, whereas the printed group showed well-organized connective tissue between the rat cranial bone and the scaffold [54]. As a fibrous connective tissue connecting the tooth root to the alveolar bone, the PDL prevents infection and bone resorption associated with mechanical stress [55] and exists to allow dynamic action even in functionally strong osseointegrated implants [56]. 3D bioprinting produces bioimplants that produce structures similar to the PDL of natural teeth, which shows the value of 3D bioprinting in reconstructing periodontal tissue and treating tooth loss. However, current research using 3D bioprinting is still in the initial stage. The technology necessary to resolve microstructures deserves further exploration, and the issues of printing accuracy, cytocompatibility, and blood supply still need to be solved.

Tissue engineering in periodontal regeneration has evolved greatly in recent years from using only one type of cell to combinations of different cell types and from single scaffold structures to the emergence of techniques that mimic periodontal tissue structures that all hold promise for achieving periodontal regeneration in the clinic (An overview of these studies is given in Table 2). Although a single-center, randomized trial that used autologous PDLSCs-based treatment for periodontal intrabony defects showed it was safe, it still needs more rigorous clinical trials to evaluate the efficacy of this therapy [57]. However, several issues still need to be addressed for stem cell transplantation: safe and effective in vitro expansion; effective transplantation methods; long-term storage of engineered tissues; standardized procedures for sterilization, purification, and the quantitative production of cell products; age limitations for autologous cell transplantation; and the potential risks and availability of allogeneic and xenogeneic cells. Further research and understanding of stem cell physiology may facilitate the development of novel and powerful therapeutic approaches and will help realize the tremendous impact of stem cell therapies on the future of health care. A prerequisite for functional periodontal tissue regeneration is novel biomaterial technology aimed at mimicking the structure of periodontal tissue closely at the micron and nanoscale. Technologies, such as electrostatic spinning and 3D bioprinting, hold promise, but more research is needed to address the need for improved cell viability, printing accuracy, etc.

**Table 2:** An overview of these studies on cell-based tissue engineering

Cells types	Delivery method	Culture conditions	Animal models	Results	References
BMMSCs	Cell suspension	Minimal medium	Rat model of periodontitis	Comparing the BMMSCs injection group to the control groups, clinical examinations, X-rays, and histological analyses demonstrated considerable periodontal tissue regeneration. The inflammatory mediators interleukin 1β (IL-1β), interferon-γ (IFN-γ), and tumor necrosis factor-α (TNF-α) were also blocked	[17]
hPDLSCs	Cell sheets	Temperature responsive dishes	CB-17/Icr-scid/scid mice	The hPDLSCs sheets exhibited no microbial contamination, strong periostin expression, and alkaline phosphatase activity. In vivo, it led immune-deficient rats to develop cementum and PDL-like tissue	[26]
Human DPSCs (hDPSCs)	Cell sheets and cell suspension	20.0 ug/ml Vc	Miniature pigs of the periodontitis	As compared to hDPSCs injection, hDPSCs sheets demonstrated a greater ability for bone repair	[20]
Rat PDL Cs and osteoblast like cells (MC3T3-E1cells)	Composite cell sheets	Temperature responsive dishes	Immunocompromised mice	In ectopic and orthotopic transplantation, a composite cell sheet containing 10 layers of cells regenerated functional PDL-like fibers and similar alveolar bone periodontal ligament structure	[28]
hPDLSCs and hJBMMSCs	Composite cell sheets	50 μg/ml L-ascorbic acid	Nude mice of ectopic transplantation	The composite cell sheets enhanced the expression of genes and proteins associated with bone and extracellular matrix	[29]
Human HUVECs and PDLCs	Composite cell sheets	Temperature-responsive culture dishes	Transplanted subcutaneously into immunodeficient mice	The cell sheets resulted in the creation of PDL-like tissues in vivo and increased numbers of blood vessel lumens while reducing necrosis and inflammation of the transplanted tissue	[30]
hPDLSCs, human alveolar bone stem cells (hABSCs) and human gingival margin-derived cells	A calcium phosphate-coated melt electrospinning polycaprolactone (CaP-PCL) scaffold that supports cell sheets	Temperature-responsive culture dishes and 50 μg/mlVc	Periodontal defect model in the athymic rat	Significant periodontal attachment formation was observed with the addition of hABSCs and sheets of hPDLCs. Human gingival margin-derived cell sheets failed to induce periodontal regeneration on the root surface as well as suppressed bone formation within the CaP-PCL scaffold The scaffold optimized the regenerative effect of tooth-derived stem cells on cementum, PDL and alveolar bone. A well aligned PDL-like collagen fibers are generated which are inserted into the cementum and alveolar bone in mice	[34]
DPSCs, PDLSCs and ABSCs	3D printing PCL/HA scaffold	Human amelogenin, and bone morphogenetic factor(BMP)-2	Transplanted subcutaneously into immunodeficient mice (Harlan)	The scaffolds optimized the regenerative effect of dental stem cells on cementum, PDL and alveolar bone. A well-aligned PDL-like collagen fibers are generated which are inserted into the cementum and alveolar bone in immunodeficient mice	[36]
Merino sheep PDLSCs and osteoblast	A biphasic scaffold based on the electrospun loaded cell sheets	Osteogenic media	The immunodeficient subcutaneous model	Higher mineralization density and improved adherence to the dentin surface could be seen in biphasic scaffolds	[37]
hPDLSCs human GMSCs human osteoblast	A trilayer porous scaffold based on chitosan—human compartments	-	Ectopic model in nude mice	Scaffolds exhibit high biocompatibility with tissue growth and vascularization in the wild-type mice and identified in nude mice a rich mineralized matrix inside the medium molecular weight-chitosan area, with weakly mineralized deposits at the dentin contact	[38]
-	A porous tri-layered scaffold	Cementum protein 1, fibroblast growth factor 2(FGF2), and platelet-rich plasma derived growth factors	Maxillary periodontal defects in rabbits	On examination of the microcomputed tomography, porous trilayer structure had fully repaired the defect and closed the wound. In contrast to the other three groups, the newly produced cementum, fibrous PDL, and alveolar bone with distinct bony trabeculae were mostly formed	[39]
rBMSCs	Porous chitosan was created using PCE copolymer electrospun nanofibrous mats	–	Sprague–Dawley rats periodontal defects	The electrospinning scaffold stimulates in vitro directed organization and ligament generation of rBMSCs. In comparison with the other two groups, it demonstrated in vivo a more regular organization of regenerated PDL, a wider development of mature collagen fibers, and an increase in periostin expression	[42]
Ovine osteoblasts	A biphasic scaffold polycaprolactone melt electrospun scaffold	–	Ectopic model in athymic nude rats	Alkaline phosphatase activity and mineralization significantly increased in the CaP-coated group, while in vivo, there was more bone formation, more pronounced vascularization, and greater periodontal attachment on the dentin surface	[43]
–	3D-printed PCL scaffolds	rhPDGF-BB	Human clinical trials	The scaffold was uncovered at 13 months; however, at 14 months, they found a 3-mm increase in adhesion and 75.9% stent retention, with primarily connective tissue healing and no bone repair evidence	[49]
hPDLSCs	Bioscaffolds made by extrusion 3D bioprinting technology	–	–	A composite of SA/Gel/n-HA hydrogels exhibited well rheological properties and a high swelling rate suitable for the printed scaffolds, which indicated that they had sufficient porosity	[51]
hPDLSCs	3D cell-printing	–	Calvarial defect model in athymic rats	As compared to the cell seeding group, the printed group showed orderly connective tissue between the scaffold and the athymic rat cranial bone	[54]

### Endogenous regeneration and periodontal regeneration

Although cell therapy has shown regenerative potential in the treatment of periodontal defects, the translation of cell therapies has been hampered by challenges in production, storage, delivery to patients, optimal cell viability, stem cell immunogenicity, ethics, and safety. Some studies [58, 59] found that in periodontal regeneration, only a small percentage of green fluorescent protein-labeled transplanted cells existed in defect areas, suggesting that the transplanted stem cells may be less engaged in differentiation to repair damaged tissue. According to previous stem cell transplantation studies, transplanted cells are believed to promote tissue regeneration through two possible pathways: participating in the formation of tissues through their own proliferation and differentiation (direct contribution) or inducing the formation of new tissues in host cells through the secretion of cytokines/growth factors (indirect contribution). A new view has emerged that MSCs may affect tissue repair primarily through their paracrine factors and the stimulation of host cells, rather than through cell replacement [60, 61].

MSCs secretome derivatives, such as conditioned medium (CM), include different proteins, cytokines, growth factors, enzymes, extracellular vesicles (EVs) [62] with various proteins, such as cargo, coding and noncoding RNA, small RNAs, autophagosomes, and mitophagosomes. EVs can be further subdivided into apoptotic bodies, microparticles, and exosomes. Subsequent studies [63] have revealed that exosomes participate in cell communication, immunomodulation, promotion of cell proliferation, angiogenesis, and neuroprotection, which largely explains the extremely broad therapeutic effects previously attributed to MSCs. Secretome derivatives are gaining popularity due to their safety, low immunogenicity, and the reduced number of ethical issues involved. Furthermore, the ECM [64] is an intricate and dynamic bioenvironment with precisely regulated mechanical and biochemical properties that provides the appropriate microenvironment for cells and acts as a powerful tool for endogenous regeneration without cellular involvement. Drugs targeting signaling molecules involved in periodontitis progression, modulating osteoimmunity and promoting tissue repair and regeneration to drive endogenous regeneration may also be used as a tool for cell-free therapy.

Strategies, such as adding biological agents (growth factors, exosomes, and CM), adding well-designed or natural biomaterials (ECM) to induce cell homing and promote cell residence and differentiation, and using signaling molecules to drive endogenous regeneration, are all based on the concept of endogenous regeneration. Endogenous regeneration [65] involves creating a microenvironment suitable for the promotion of cell proliferation and differentiation and mobilizing chemokines and cytokines to promote the continuous migration and aggregation of the organism's internal stem cells to the injured region. This section describes the current state of various strategies for endogenous regeneration (Studies of this section is summarized in Table 3).

**Table 3:** Various strategies for endogenous regeneration in periodontal regeneration

Cell-free therapeutics	Scaffolds types	Study mode	Results	References
Stromal cell derived factor-1 (SDF-1) and BMP-2	Supramolecular	Rats with a maxillary periodontal bone deficiency	The release of these two bioactive substances from the hydrogel was precise, synchronized, and continuous, according to in vitro and in vivo data. And a better bone regeneration rate of 56.7% bone volume fraction was attained	[68]
CEMP1, FGF 2, platelet-rich plasma-derived growth factors	A scaffold made of trilayered nanocomposite hydrogel	Maxillary periodontal defects in the rabbits	Further evidence of the formation of new cementum, fibrous PDL, and alveolar bone with distinct bony trabeculae in contrast to those of the other three groups obtained from histological and immunohistochemical analyses	[39]
Vascular endothelial growth factor (VEGF) and BMP-2	Gelatin microparticles incorporated within the scaffold pores	Rat cranial critical size	Large quantities of bone growth were seen in the scaffold pores and along its outer surfaces in the dual release and BMP-2 groups. Osteoid secretion and mineralization were also noticeable, and new bone was frequently in close or direct contact with the scaffold interface. At 4 weeks, there was no discernible difference in blood vessel creation across the groups	[70]
VEGF,FGF2 and BMP-2	A biomimetic electrospun nanocomposite fibrous scaffold	Wistar rats	Although FGF2 and VEGF loaded scaffolds had a varied release pattern, both of the dual growth factor loaded scaffolds (VEGF + BMP-2/FGF2 + BMP-2) improved vascularization and new bone production	[71]
CM obtained from cultured PDLSCs	–	A rat periodontal defect model	TNF-mRNA levels in healing periodontal tissues were reduced as a consequence, while those in the IFN-stimulated monocyte/macrophage cell line RAW were inhibited	[77]
CM from GMSCs and PDLSCs	Collagen membranes loaded with concentrated CM	A rat periodontal defect model	They both enhanced periodontal regeneration Immunostaining results showed that TNF-α and IL-1 expression levels were reduced by the CM transplantation whereas IL-10 expression levels were elevated	[78]
Human GMSCs-derived exosomes	Local injection	Ligature-induced periodontitis model in mice	Exosomal miR-1260b was reported to target the Wnt5a-mediated RANKL pathway and limit its osteoclastogenic activity in GMSCs-derived exosomes, which showed anti-osteoclastogenic acts	[79]
Human MSCs exosome	Collagen sponges	SD Rat periodontal defect model	It could promote migration and proliferation by activating prosurvival AKT and ERK signaling via CD73, which results in the regeneration of periodontium	[81]
SHED-derived exosome	–	In vitro	According to the results, Wnt3a and BMP-2 were carried by the conditioned SHED-Exo-increased PDLSCs, which led to their accelerated osteogenic differentiation	[82]
SHED-derived exosomes	β-TCP	Rat models of the periodontal defect	Through the modulation of angiogenesis and osteogenesis, the exosomes/β-TCP group aids in the healing of alveolar bone defects	[83]
Decellularized tooth bud-ECM structures	–	Extraction sockets of adult piglets	It was discovered that the complex structure regularly directed the creation of well-organized bioengineered teeth with dimensions similar to those of natural human teeth	[87]
Decellularized PDLSCs sheets	PCL	A rat periodontal defect model	Decellularized PDLSCs sheets could promote angiogenesis and increase periodontal attachment formation	[88]
Decellularized human PDLSCs sheets	PCL/gelatin nanofibers	A rat periodontal defect model	It leaded to bone, dental bone, and periodontal ligament formation after 4 weeks	[89]
Sclerostin-neutralizing monoclonal antibody (Scl-Ab)	Administered subcutaneously and locally	The experimental periodontitis rats model	In terms of linear alveolar bone loss, volumetric measures of bone support, such as bone volume fraction and tissue mineral density, as well as higher levels of the serum procollagen type I amino-terminal propeptide and bone-formation markers osteocalcin, maxillary bone healing was significantly improved after 6 weeks of Scl-Ab	[97]
Sclerostin antibody (SAB) alone and DKK1 antibody (DAB)	Systemic administration	A chronic rat maxillary molar extraction model	Additionally, because of hypo-occlusion and increased alveolar bone density, the opposing mandible experienced bone loss that SAB and SAB + DAB completely stopped	[98]
Complement, C3 inhibitor (CP40)	Locally administration	Nonhuman primates and C3-deficient or wild-type mice	When compared to a control therapy, local Cp40 administration prevented ligature-induced periodontitis and bone loss and lowered osteoclastogenesis in bone biopsy specimens in nonhuman primates	[101]

### Scaffold-based cell-free therapy

Several events need to occur in order to repair periodontal defects caused by periodontitis or other causes, including cell proliferation, migration differentiation and angiogenesis. Particular factors participating in these processes will also be needed, such as SDF-1, BMP, insulin-like growth factor (IGF-1), and tumor necrosis factor β1 (TGF-β1) [66–68]. Bioactive agents can be added into biomaterials by noncovalent methods such as physical capture, surface adsorption, and ion complexation [67].

However, a single growth factor has a limited ability to regenerate tissue, and an overdose of a single growth factor can cause severe inflammatory reactions [69]. Several studies revealed that the use of both angiogenic and BMP cytokines was more effective than the use of one single growth factor [70, 71]. Cytokines are mainly large molecular proteins with a short biological half-life, poor stability, and high cost, but these deficits can be overcome by modifying and packaging cytokines with scaffold materials for spatiotemporal control. Chitosan/alginate/PLGA hybrid scaffolds were designed by Duruel et al. for the coordinated and sequential administration of IGF-1 and BMP-6 [72]. Cell culture studies showed that the scaffolds induce the proliferation and osteoblastic differentiation of cementoblasts more effectively than IGF-1- and BMP-6-free chitosan scaffolds. Tan et al. [68] designed a hydrogel called NapFFY that allows the release of two bioactive factors, SDF-1 and BMP-2, from the hydrogel in a controlled, sequential and sustained manner as intended. These conditions were created by using various materials in combination with growth factors to create favorable alveolar bone, PDL, and alveolar bone growth conditions. Cellular studies showed that the scaffold aided in the differentiation of human DFCs into cementogenic, fibrous, and osteogenic tissues, and in vivo findings further supported the formation of new cementum, fibrous PDL, and alveolar bone with distinct bone trabeculae. [39].

Several growth factors are effective for tissue reconstruction in specific conditions but are not sufficient for periodontal tissue regeneration processes that involve multiple growth factors and in which diverse types and volumes of factors are involved at different times. In this way, MSCs-CM or EVs offer new possibilities.

### MSCs-conditioned medium and exosomes

In recent years, MSCs-CM and exosomes have been widely used in the field of skin, cardiovascular, liver and kidney tissue regeneration due to their angiogenic, anti-inflammatory, immunomodulatory, and anti-apoptotic activities as well as their cell growth support and chemoattraction [73, 74]. Their use in periodontal tissue regeneration is gradually increasing. MSCs-CM [61] contains anti-inflammatory TGF-β1, IL-10, IL-12, IL-17E, IL-27, and proinflammatory cytokines, such as IL-1b, IL-6, IL-8, and IL-9. The final outcome can depend on how these proinflammatory and antiinflammatory cytokines are balanced. Reduced salivary CD9/CD81 exosome levels were linked to the pathogenesis of periodontitis, according to a research article [75]. In periodontitis, programmed death-ligand 1 (PD-L1) expression positively correlated with inflammation, whereas PD-L1 mRNA in salivary exosomes was enriched [76]. This finding suggests that exosomes may be involved in inflammatory signaling and periodontal disease progression. Nagata et al. [77] showed that transplantation of CM obtained from cultured PDLSCs resulted in decreased TNF-α mRNA levels in healing periodontal tissues and suppressed TNF-α mRNA levels in the monocyte/macrophage cell line RAW stimulated with IFN-γ. Qiu et al. [78] revealed that CM of both GMSCs and PDLSCs promoted periodontal regeneration in a rat periodontal defect model. Immunostaining results showed that the CM transplants decreased the expression levels of IL-1 and TNF-α and increased the levels of IL-10. Another study [79] found that TNF-α isolated from pretreated GMSCs exosomes induced polarization of anti-inflammatory M2 macrophages through the improved secretion of GMSCs exosomes and increased exosomal expression of CD73. This finding suggests that CM from PDLSCs and GMSCs has the potential to improve the microenvironment of periodontal regeneration by participating in inflammatory regulatory responses.

CM and exosomes act as carriers of growth factors and cytokines, including IGF-1, TGF-β1, VEGF, and hypoxia inducible factor-1α. These growth factors and cytokines also contribute to the recruitment of bone and vascular precursor cells, stimulate their proliferation and differentiation into osteoblasts and enhance angiogenesis, which are all key components of periodontal regeneration [80]. This notion has been validated by some studies. Human MSCs exosome-loaded collagen sponges could increase migration and proliferation through CD73-mediated adenosine receptor activation of prosurvival AKT and ERK signaling, which leads to the regeneration of periodontal tissues [81]. A study [82] indicated that conditioned SHED exosome-enhanced PDLSCs osteogenic differentiation was partly due to it carrying Wnt3a and BMP-2. Exosomes released by human SHED, according to a study from Wu et al., support the healing of alveolar bone defects by controlling angiogenesis and osteogenesis [83]. In other fields of tissue regeneration, MSCs-derived exosomes have been found to act mainly through the horizontal transfer of mRNA, miRNA, and proteins, which then alter the activity of target cells through various mechanisms [84]. The mechanisms by which exosomes promote tissue regeneration are diverse and complex, and this feature may be partly due to the large number of components they contain.

Investigating the mechanisms by which exosomes promote tissue regeneration and repair would contribute to a better understanding and use of MSCs therapy. Although a substantial number of studies have reported that exosomes from MSCs are as effective as MSCs in preserving tissue and/or promoting functional recovery after injury, it is difficult to compare efficacy due to the lack of appropriate controls, differing doses, the use of different disease endpoints, and the frequency and duration of doses. There is also the question regarding whether exosome number, instability, and freezing/thawing affect exosome potency. In addition, the status of MSCs-derived exosomes depends on the cellular origin and physiological state with different cells and different states having different effects. Finally, the standardization of production and storage of the product also requires further investigation.

### Extracellular matrix

Natural tissues are a mixture of cells, growth factors, and ECM. Unlike CM or exosomes, the ECM [85] is an intricate and dynamic bioenvironment with precisely regulated mechanical and biochemical properties, providing the appropriate microenvironment for cells, influencing stem cell behavior, and participating in mechanical signaling through mechanoreceptors (e.g., integrins). ECM extraction from natural tissues is accomplished by a decellularization process that uses chemical, enzymatic, and/or physical means to remove cellular and genetic material from ECM that would cause immunogenicity while maintaining its structure, including proteins and polysaccharides. Thereafter, the decellularized ECM can be populated with the patient's cells to produce individualized tissue [64]. Decellularized ECM have been used to recreate skeletal muscle, spinal cord, bone, kidney, liver, lung, and heart tissue [86].

Tissue-derived ECM have some complex structures, some of which are difficult to separate, so incomplete decellularization occurs and creates the risk of immune rejection and disease transmission. Mixing different cell types to create tissue scaffolds can address some of the limitations of tissue-derived ECM. Selecting appropriate cells to secrete and integrate new components to form an ECM allows for their further modification (e.g., genetic) and exposes them to specific stimuli, thus enabling the creation of an ECM with desired properties. Combining porcine dental epithelial cells with human DPSCs and HUVECs and implanting them into extraction sockets of adult piglets led to decellularized tooth bud-ECM structures, which were found to consistently guide the formation of organized bioengineered teeth comparable in size to natural human teeth [87]. Decellularized PDLSCs sheets have been coupled with PCL scaffolds to promote angiogenesis and increase periodontal attachment formation [88]. Another study used decellularized PDLSCs sheets with PCL/GE nanofibers as carriers of 15-deoxy-delta(12,14)-prostaglandin J(2) nanoparticles. These nanoparticles were transplanted into periodontal defects in rats, leading to bone, dental bone, and periodontal ligament formation after 4 weeks [89]. The decellularized ECM scaffold was approved recently by the US FDA for human therapeutic applications, making ECM scaffolds a promising therapy for human tooth regeneration.

The most important aspects of the ECM are its intrinsic properties, including both biochemical and biomechanical properties. However, current methods are still some distance from this goal to achieve a complete intrinsic vital structure, but the process of producing ECM scaffolds can be modified by adding growth factors and bioactive molecules to the ECM. Combinations of different techniques for different tissues can improve the efficiency of decellularization and reduce the negative effects caused by using a single technique. Optimizing decellularization methods to utilize scaffolds from human and animal tissues and organs can help address the increasing needs of patients for clinical transplantation.

### Drug therapy

Periodontitis is a disease caused by the destruction of periodontal tissue when the inflammatory response, wherein plaque serves as the initiating factor, exceeds the host's immune regulatory capacity. The conventional approach to inflammation management is surgical and nonsurgical treatment coupled with antibiotics as an adjunct to therapy, but there are limitations in drug resistance and effectiveness. Alternative therapies that target the signaling molecules that participate in the progression of periodontitis, modulate osteoimmunity and promote tissue repair and regeneration have emerged.

Teriparatide (FORTEO) [90] is an anabolic drug that consists of the bioactive portion of parathyroid hormone which activates a signaling cascade to stimulate bone formation. Clinical studies involving the systematic administration of teriparatide for periodontal tissue regeneration showed that it had a significant bone formation effect [91], which is consistent with the results of preclinical studies [92].

Sclerostin and Dickkopf-related protein 1 (DKK-1) are both antagonistic regulators of the Wnt signaling pathway that limit bone growth [93]. These proteins at higher levels in patients with periodontitis than in patients without periodontitis [94] and are expressed at significantly higher levels in rats with experimental periodontitis [95]. Romosozumab, blosozumab and BPS-804 (setrusumab) inhibit sclerostin, of which romosozumab has been approved for marketing by the FDA. Previous studies showed that a Scl-Ab increased bone mass and bone density and decreased bone resorption [96]. Taut et al. [97] discovered that 6 weeks of Scl-Ab significantly improved maxillary bone healing, as measured by linear alveolar bone loss, bone volume fraction, and tissue mineral density. Another study [98] showed that scl-Ab combined with DKK-1 antibody more dramatically enhanced alveolar process healing. As mentioned before, these studies were undertaken by means of systemic administration, which suffers from safety and unclear tissue localization. These issues can be solved by local administration. In a study comparing the effectiveness of systemically and locally administered low-dose Scl-Ab using PLGA microspheres in rats with periodontitis, it was found that systemic Scl-Ab significantly enhanced alveolar bone and cementum regeneration in comparison to locally administered Scl-Ab [99]. This finding can be attributed to the difference in dosage and characteristics of the PLGA microspheres. Consequently, for local drug administration, various factors, such as the form of the drug, the mode of transport, and the spatiotemporal controlled release of the drug, need to be addressed in a multidisciplinary manner.

Currently, the complement system is considered a pivotal immune locus for inducing and regulating various immune and inflammatory functions [100]. Studies have demonstrated that the complement system is overactivated during periodontitis and that AMY-101 (C3 inhibitor) targets its complement component (C3) with therapeutic benefit and no adverse toxicity [101]. Preclinical trials [102] and a phase IIa clinical study that was randomized, placebo-controlled, and double-blind (NCT03694444) confirmed that C3-targeted inhibition prevented gingival inflammation in patients with periodontitis. C3-targeted intervention may represent an innovative host-modulated therapy in the treatment of periodontitis.

### Combination of MSCs secretome derivatives with MSCs

The ability of MSCs to multi-differentiate is key to their role, but maintaining the integrity of MSCs in the microenvironment of the transplanted region is a great challenge. Exosomes and ECM, which contain a variety of bioactive molecules, regulate functions such as inflammatory response, signal transduction, and maintenance of cell function and structure [62]. Many studies have been conducted on the application of exosomes in periodontal regeneration, but the use of cell derivatives in combination with MSCs for periodontal regeneration has not been reported. However, there are some reports of other tissue regeneration. After rabbit cartilage progenitor cell-alginate constructs were implanted subcutaneously in nude mice, the injection of exosomes derived from chondrocytes weekly might imitate the chondrogenic niche, which maintenance of cartilage stability [103].

The method for creating chondrocytes folded within hBMSCs-ECM requires significantly fewer cells than the chondrocyte cell sheet technology, and it also exhibited chondrocyte proliferation, maintenance of chondrocytic phenotype, and promotion of chondrocyte redifferentiation after expansion both in vitro and in vivo [104].The ECM derived from adipose tissue has basic components of the gradient tendon-to-bone-interface (TBI)structure, after adding the HA gradient to the scaffold, which could provide umbilical cord derived MSCs with a favorable microenvironment to regenerate the structure of the TBI [105]. The multilayered interconnected structure of periodontal tissue is similar to that of TBI, and the microenvironment provided by the extracellular matrix that favors MSCs in combination with dental stem cells may also help to reconstruct the ordered structure of the periodontium.

LL37, an antimicrobial peptide, plays a central role in wound healing, angiogenesis, and arteriogenesis and acts as an immune adjuvant [106]. One study discovered that the combination of LL37 and BMP2-modified MSCs promoted MSC-mediated increaseed the number of new blood vessel and ectopic bone formation in the LPS-induced osteolytic defects in mouse calvaria. This hints to us that LL37 can be a potential candidate drug for periodontal regeneration because of its ability to promote osteogenesis and for inhibiting bacterial growth [107].

Combining MSCs derivatives with MSCs is more effective and maximizes the potential of MSCs, but the difficulty of establishing standardized procedures for exosome and MSC isolation, purification, storage, and standardization increases accordingly. Clinical treatment plans are not static due to the diversity of the periodontal foundation of the patients, and cell and cell free therapy each have their own advantages and limitations, and either alone or in combination are applied to specific type. Further studies on the identification, signal transduction, and mechanisms of action of key active molecules in MSCs, exosomes, and ECM are needed.

## Conclusion and future perspectives challenge

MSCs-based periodontal tissue engineering applications, especially the construction of a microenvironment that is conducive to periodontal regeneration through well-designed guided scaffold structures that are biomimetic of periodontal multilayer structures, have made promising progress. However, the application of MSCs still faces numerous challenges, including the safety of cell expansion in vitro, standardized production procedures for cellular products, inherent immunogenicity, precision of scaffold materials, cytocompatibility, and material degradation. Cell-free therapy with the addition of biological agents (growth factors, exosomes, and CM) to promote endogenous regeneration in organisms has partially addressed the limitations of cell therapy and contributed to understanding the mechanisms behind MSCs functions. However, the environment and state of exosome-derived cell culture strongly influence the properties of exosomes. Defining exosome activity, dose, and potency as well as effective methods of extraction and storage require additional studies. Finally, drug therapy targeting signaling molecules aids in functional periodontal regeneration.

## Data Availability

Not applicable.

## Abbreviations

PDL: Periodontal ligament
MSCs: Mesenchymal stem cells
DPSCs: Dental pulp stem cells
PDLSCs: Periodontal ligament stem cells
SHED: Stem cells from human exfoliated deciduous teeth
GMSCs: Gingival MSCs
BMMSCs: Bone marrow MSCs
ECM: Extracellular matrix
HUVECs: Human umbilical vein endothelial cells
HA: Hydroxyapatite
PLGA: Poly(lactic-co-glycolic acid)
PCL: Polycaprolactone
3D: Three-dimensional
β-TCP: β-Tricalcium phosphate
PCE: Poly(caprolactone)-poly(ethylene glycol)
CaP: Calcium phosphate
rhPDGF-BB: Recombinant human platelet-derived growth factor-BB
hPDLSCs: Human PDLSCs
Gel: Gelatin
na-HA: Nano-hydroxyapatite
SA: Sodium alginate
CEMP1: Cementum protein1
TNF-α: Tumor necrosis factor-α
IFN-γ: Interferon-γ
IL: Interleukin
hDPSCs: Human dental pulp stem cells
hJBMMSCs: Human jaw bone marrow mesenchymal stem cells
hABSCs: Human alveolar bone stem cells
BMP: Bone morphogenetic factor
FGF2: Fibroblast growth factor
rBMSCs: Rat bone marrow mesenchymal stem cells
CM: Conditioned medium
EVs: Extracellular vesicles
SDF-1: Stromal cell derived factor-1
IGF-1: Insulin-like growth factor-1
TGF-β1: Tumor necrosis factor β1
hBMGCs: Human bone marrow mesenchymal stem cells
PD-L1: Programmed death-ligand 1
DKK-1: Dickkopf-related protein1
Scl-Ab: Sclerostin-neutralizing monoclonal antibody
SAB: Sclerostin antibody
DAB: DKK1 antibody
TBI: Tendon-to-bone-interface
